# Noninvasive ventilation as the first choice of ventilatory support in children

**DOI:** 10.5935/0103-507X.20190045

**Published:** 2019

**Authors:** Aline Rafaele Barros da Silva Lins, Maria do Carmo Menezes Bezerra Duarte, Lívia Barboza de Andrade

**Affiliations:** 1 Unidade de Terapia Intensiva Pediátrica, Hospital Esperança Recife- Recife (PE), Brasil.; 2 Programa de Pós-Graduação, Instituto de Medicina Integral Prof. Fernando Figueira - Recife (PE), Brasil.

**Keywords:** Noninvasive ventilation, Child, Intensive care units, pediatric, Respiratory insufficiency, Comorbidity, Ventilatory support

## Abstract

**Objective:**

To describe the use of noninvasive ventilation to prevent tracheal intubation in children in a pediatric intensive care unit and to analyze the factors related to respiratory failure.

**Methods:**

A retrospective cohort study was performed from January 2016 to May 2018. The study population included children aged 1 to 14 years who were subjected to noninvasive ventilation as the first therapeutic choice for acute respiratory failure. Biological, clinical and managerial data were analyzed by applying a model with the variables that obtained significance ≤ 0.20 in a bivariate analysis. Logistic regression was performed using the ENTER method. The level of significance was set at 5%.

**Results:**

The children had a mean age of 68.7 ± 42.3 months, 96.6% had respiratory disease as a primary diagnosis, and 15.8% had comorbidities. Of the 209 patients, noninvasive ventilation was the first option for ventilatory support in 86.6% of the patients, and the fraction of inspired oxygen was ≥ 0.40 in 47% of the cases. The lethality rate was 1.4%. The data for the use of noninvasive ventilation showed a high success rate of 95.3% (84.32 - 106). The Pediatric Risk of Mortality (PRISM) score and the length of stay in the intensive care unit were the significant clinical variables for the success or failure of noninvasive ventilation.

**Conclusion:**

A high rate of effectiveness was found for the use of noninvasive ventilation for acute episodes of respiratory failure. A higher PRISM score on admission, comorbidities associated with respiratory symptoms and oxygen use ≥ 40% were independent factors related to noninvasive ventilation failure.

## INTRODUCTION

Respiratory diseases are very common reasons for admissions in pediatric intensive care units (ICUs) in Brazil.^(1,2)^ Among such diseases, acute respiratory infections are among the major causes of morbidity and mortality in pediatric patients and are responsible for approximately 20 to 30% of deaths, especially in children under 5 years of age for whom the most frequent causes of death are pneumonia and bronchiolitis.^(3,4)^

Among the critically ill pediatric patients hospitalized in ICUs, 30 to 50% require some type of mechanical ventilation support.^(5)^ Accordingly, immediate intervention for acute respiratory failure is needed, and mechanical ventilation is the most widely used therapeutic support modality. Currently, studies suggest that noninvasive ventilation (NIV) can be implemented as the first choice for ventilatory support in select cases to obviate invasive mechanical ventilation (IMV) via orotracheal intubation.^(6,7)^

Compared to IMV, NIV has advantages, such as a lower risk of pneumonia associated with mechanical ventilation, upper airway trauma and postextubation vocal cord dysfunction, maintenance of patient communication and feeding, and a reduced need for sedation, thus resulting in a lower risk of acquired muscle weakness and a shorter duration of mechanical ventilation.^(8-10)^

NIV is being considered in many centers as an initial form of ventilatory support for acute respiratory failure in infants and children.^(11-13)^

Despite the increasing use of NIV, this therapy may not have the expected outcome in some conditions, and the need for intubation and the consequent use of invasive ventilation up to 72 hours after NIV suspension are defined as failure of NIV.^(14,15)^ Patients selected for NIV as first-line treatment to avoid invasive ventilation should show marked improvement approximately 1 - 2 hours after the onset of ventilation, especially in oxygenation-related variables.^(16,17)^

In 2017, the Pediatric Mechanical Ventilation Consensus Conference (PEMVECC) reported that the use of NIV has increased in acute respiratory infection cases, postcardiac surgery, status asthmaticus cases, and cases of neuromuscular disease exacerbations. In addition, to avoid delayed tracheal intubation, the success of NIV must be evaluated up to one hour after its initiation by observing the following parameters: heart rate, respiratory rate, the relationship between blood oxygen saturation and the fraction of inspired oxygen (SpO_2_/FiO_2_), pH, the level of consciousness and the presence of organ failure.^(18)^

The objective of this study was to describe the use of NIV to prevent tracheal intubation in children in a pediatric ICU and to identify the independent factors related to NIV failure.

## METHODS

A retrospective cohort observational study was conducted with information extracted from the management database of the pediatric ICU of the *Hospital Esperança Recife* (*Rede D'Or, São Luiz*) for the period from January 2016 to May 2018. This pediatric ICU includes ten beds available to clinical and surgical patients. The hospital is a reference hospital of medium and high complexity that has international standards accreditation (QmentumDiamante).

This study was approved by the Research Ethics Committee on Humans of the *Instituto de Medicina Integral Prof. Fernando Figueira* (IMIP) under number CAAE 90283018.4.0000.5201.

The population eligible for the study consisted of all children older than 1 month and up to 14 years and 11 months old receiving NIV as the first therapeutic choice for acute respiratory failure who were hospitalized in the unit during the analyzed period; the population was a convenience sample. Children receiving NIV at home due to chronic disease, those who received NIV only at the time of extubation, and those who arrived from the surgical block in the immediate postoperative period were excluded from the study.

After application of the eligibility criteria, biological and clinical data such as age, gender, weight, nutritional status and risk, the total number of admissions to the pediatric ICU, clinical diagnosis, the severity score at admission (Pediatric Risk of Mortality - PRISM II), the length of stay in the ICU, the total duration of NIV in days, success or failure of NIV, the mean duration, and the use rate of NIV, were collected. The variables related to the study were directly collected from data already recorded for quality management of the pediatric ICU and were recorded in a specific data sheet prepared for this study.

In the pediatric ICU, we have invasive ventilation ventilators microprocessed by NIV software and specific NIV apparatuses. Oronasal (facial) and nasal devices were used, and the choice of device was dependent on the condition and age of the child.

We considered NIV failure when a child required an artificial airway and conversion to IMV at some point during pediatric ICU hospitalization regardless of the duration of NIV.

### Statistical analysis

Statistical Package for Social Science (SPSS) version 13.0 for Windows and Excel 2010 were used for the analysis. The results are shown in tables with their respective frequencies and measures of central tendency and dispersion. The Kolmogorov-Smirnov normality test was used for quantitative variables.

A comparison of biological and clinical variables between two groups (NIV success and failure) was performed using Student's *t*-test and the Mann-Whitney test.

To compose the model, the variables that obtained significance ≤ 0.20 in the bivariate analysis were included, and a subsequent logistic regression was performed using the ENTER method. A significance level of 5% was adopted.

## RESULTS

**Figure 1:**
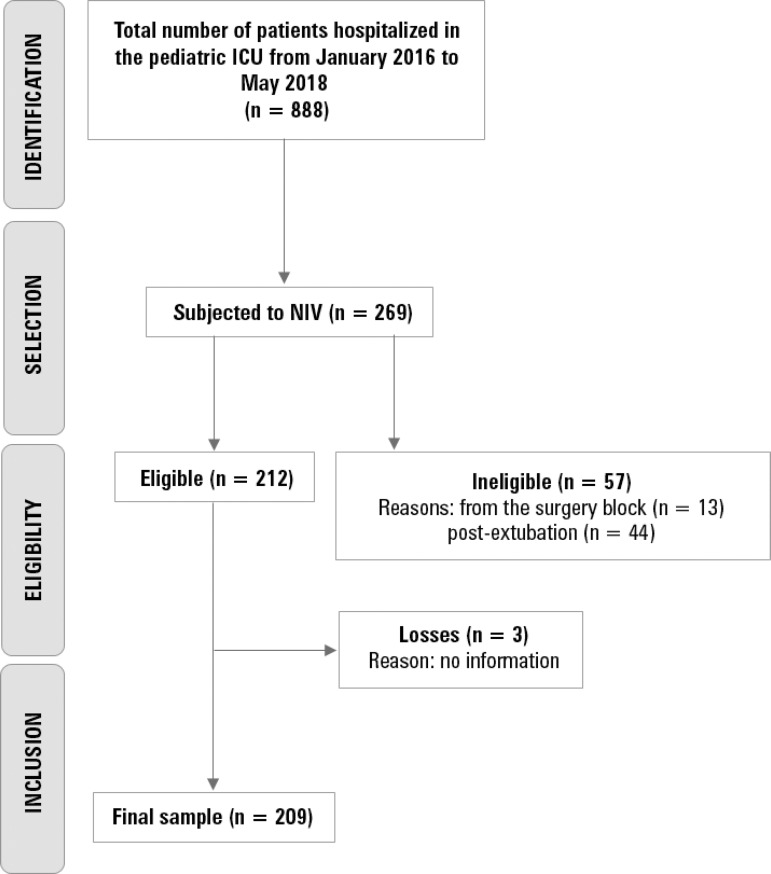
Flowchart of participant selection according to the model suggested in the STROBE statement.^(19)^

Of the 888 children and adolescents admitted to the pediatric ICU during a period of 29 months (January 2016 to May 2018), 212 were subjected to NIV, although 3 patients were lost, resulting in 209 (23.5%) children and adolescents with NIV as the first therapeutic choice (Figure 1). Information regarding the three lost patients was not found in the managerial data and was therefore included in the sample loss related to the study.

The sample consisted of children with a median age of 65 months (interquartile range, 34 - 97), 58% were male, 96.6% had respiratory disease as a primary diagnosis, and 15.8% had associated comorbidities, such as cerebral palsy, genetic syndromes or some degree of delayed neuropsychomotor development. Among the 209 patients, NIV was performed as the first ventilatory support option within the first 24 hours of hospitalization in the pediatric ICU in 86.6% of the patients, and an FiO_2_ ≥ 0.40 was used in 47% of cases. The mortality rate was 1.4%. Data on the biological and clinical variables serving as the main reason for admission to the pediatric ICU, clinical severity scores, the mean duration of ICU hospitalization, the mean duration of NIV, the NIV success rate and ICU outcomes are shown in table 1.

**Table 1:** Biological and clinical characteristics of 209 pediatric patients undergoing noninvasive ventilation

Variables	Observed values
Age (months)	65 (34 - 97)
Male gender	121 (58)
Weight (kg)	20 (14 - 29.7)
Main reasons for admission	
Asthma	81 (38.8)
Pneumonia	48 (23)
Asthma + pneumonia	46 (22)
Bronchiolitis	11 (5.3)
Acute wheezing	9 (4.3)
Other	14 (6.6)
Year of admission	
2016	293 (37)
2017	394 (44.4)
2018	201 (22.6)
PRISM	0 [0 - 20]
Nutritional status†	
Very low weight/low weight	10 (4.8)
Eutrophic	151 (72.2)
Overweight/obese	48 (23)
Nutritional risk (STRONGkids)	
Low	71 (34)
Moderate	133 (63.6)
High	5 (2.4)
NIV start time	
< 24 hours after admission	181 (86.6)
> 24 hours after admission	28 (13.4)
Mean duration of ICU stay (days)	6.3 ± 8.7
Average NIV duration (days)	3 (2 - 5)
NIV outcome	
Success	196 (93.8)
Failure	13 (6.2)
Outcome of the patient	
Discharge	206 (98.6)
Death	3 (1.4)

PRISM - Pediatric Risk of Mortality; STRONGkids - Screening Tool Risk Nutritional Status and Growth; NIV - noninvasive ventilation; ICU - intensive care unit.

† according to the Ministry of Health criteria. The results are expressed as the median (minimum and maximum values), percentage n (%) or median [interquartile range].

The data for NIV use, including the use rate and success rate, the duration of NIV, and the number of patients-day who used this type of ventilatory support, are shown in table 2.

**Table 2:** Data for noninvasive ventilation use

Variables	2016 - 2018 (N = 209)
NIV success rate (%)	95.3 [84.32 - 100]
NIV use rate as a first option (%)	23.2 [20 - 26.32]
Mean NIV duration (days)	3 (2 - 5)
Patient-day who used NIV during ARI	28 ± 19.7
Patient-day in the pediatric ICU	256 ± 28.5

NIV - noninvasive ventilation; ARI - acute respiratory infection; ICU - intensive care unit. The values are expressed as the median [interquartile range] or the mean (standard deviation). *T*-test, p < 0.05.

Table 3 shows the comparison of clinical variables of children who experienced NIV success or failure. A significant difference was observed only in the PRISM II score.

**Table 3:** Comparison of clinical variables in relation to noninvasive ventilation success or failure

Variables	Success		p value
	Yes (n = 196)	No (n = 13)	
	Mean ± SD	Mean ± SD	
Age (months)	68.61 ± 41.95	71.69 ± 50.26	0.800*
Weight (kg)	22.84 ± 12.70	25.72 ± 15.02	0.493†
PRISM	0.73 ± 1.41	8.08 ± 7.03	< 0.001†
Tniv (days)	3.73 ± 4.69	5.23 ± 9.73	0.352†

SD - standard deviation; PRISM - Pediatric Risk of Mortality; Tniv - duration of noninvasive ventilation.

* Student's *t*-test;

† Mann-Whitney test.

According to the logistic regression analysis shown in table 4, the use of oxygen ≥ 40% and the presence of comorbidities were significant for NIV failure. In addition, the presence of comorbidities associated with the respiratory condition at admission increased the risk of NIV failure 14.59-fold.

**Table 4:** Multivariate regression analysis of factors related to failure of noninvasive ventilation used in children as the first choice for ventilatory support

Variables	OR	OR 95%CI	p value
Nutritional status - normal			
Yes	1.00	-	0.558
No	1.46	0.41 - 5.23	
Final model			
Oxygen use higher than 40%			
Yes	10.75	2.07 - 55.95	0.005
No	1.00	-	
Comorbidity			
Yes	12.57	3.42 - 46.24	< 0.001
No	1.00	-	

OR - odds ratio; 95%CI - 95% confidence interval. Variables that started in the model: oxygen use higher than 40%, comorbidity and nutritional status. Logistic regression model using the ENTER method.

## DISCUSSION

The use of NIV in clinical practice as the first option for ventilatory support to avoid endotracheal intubation and IMV has increased worldwide. In our study, we observed a high rate of NIV effectiveness as indicated by NIV success in 95.3 [84.32 - 106] children who received treatment for acute episodes of respiratory failure. This value may be explained by the early initiation of NIV in the pediatric ICU; 86.6% of the children started NIV within the first 24 hours after admission. The ICU provided 24-hour physical therapy with two professionals each morning, which enabled bedside assessments of the critically ill children.

Less expressive results were observed in a study conducted in a pediatric ICU in Turkey from 2012 to 2014,^(20)^ which evaluated 160 cases of NIV use for acute respiratory infection. Among these cases, NIV was used as the first support option in 89 cases, resulting in a 74.2% NIV success rate and a 25.8% NIV failure rate. The failure rate in the present study was only 6.2% (13 children among a total of 209).

In a 7-year retrospective study published in 2011, James et al.^(21)^ found an NIV success rate of 64% (53 of the 83 patients who used NIV as their first option), which was related to the prevention of intubation. The authors also reported that the oxygen support before starting NIV and its maintenance at high levels after 2 hours were higher in patients whose therapy failed, suggesting that higher levels of oxygen therapy can predict NIV failure. Similarly, regarding oxygen support, an FiO_2_ ≥ 40% was associated with a statistically significant increased risk for NIV failure.

According to our findings, the mean length of ICU stay among children who underwent NIV was 6.3 ± 8.7 days. Moreover, the group that achieved success showed a significant reduction in the length of stay in the ICU. In a study with adults, Pallin et al.^(22)^ analyzed three groups (control, NIV and IMV), and the length of hospital stay in the NIV group was higher than that in the control group but lower than that in the group receiving invasive ventilation; however, the patients who underwent NIV represented a cohort with more severe acute diseases.

According to Izquierdo et al.,^(23)^ 70.2% of 252 patients experienced successful NIV. NIV failure predictors included a partial pressure of carbon dioxide (PaCO_2_) > 35mmHg, a partial pressure of oxygen (PaO_2_) < 60mmHg and a PRISM II score at admission > 10. In our research, blood gas data could not be analyzed; however, a relationship was found between the highest severity score (PRISM) and NIV failure. Similar data were also found in 2015 and 2016 by Bakalli et al.^(24,25)^ who reported that a PRISM score below 10 points may predict a lower risk of NIV failure. Yaman et al.^(20)^ also found that the highest PRISM II score is an independent variable that can predict the risk of NIV failure.

The rate of NIV use as the first choice of ventilatory support in acute respiratory infection cases was 23.2 [20 - 26, 32], suggesting that in the pediatric ICU, NIV is most often performed preventively to avoid the use of invasive ventilation. No publications on the rate of NIV use were found in the pediatric literature.

The mean NIV duration was 3.5 days. Yaman et al.^(20)^ found a median NIV duration of 48 hours in the studied groups.

In a study conducted in pediatric ICUs in the United Kingdom and Ireland, Morris et al.^(26)^ concluded that NIV use compared with IMV use as a first-line therapy was associated with significant decreases in mortality, the length of stay in the ICU and the NIV duration. They also observed that the first-line NIV use rate was 23.2 ± 2.3% among the total number of children admitted to the unit, and the NIV failure rate was 25.7%. Our results show that the mean length of stay in the pediatric ICU was shorter for patients who experienced NIV success (4.81 ± 5.06 days) than that for patients who had NIV failure (28.08 ± 29.00 days). Our rate of failure of NIV as the first treatment option was only 6.2%.

When NIV fails, the patient should be addressed promptly. Mortality is higher in patients who transition from NIV to IMV, and a late indication to transition from NIV to IMV may contribute to this outcome.^(23-27)^ While the failure rate (6.2%) found in this study was low, three deaths were also noted - two of which occurred in the group with NIV failure. Similar data were found by Yaman et al.,^(20)^ with 7 deaths in the group with NIV failure and 1 death in the group with NIV success.

NIV failure can be categorized.^(27)^ Of the 13 cases of failure in this study, 7 (53.8%) can be categorized as intermediate failure, and 6 (46.2%) can be categorized as late failure. Yaman et al.^(20)^ found 8 children with failure between 2 and 6 hours of NIV, 18 children with failure between 6 and 24 hours of NIV, and 22 children with failure after 24 hours of NIV.

The current study found that the presence of comorbidities (mostly neurological disorders) associated with respiratory symptoms on admission increases the risk of NIV failure. Corroborating these results, Morris et al.^(26)^ compared two groups receiving invasive and noninvasive ventilation and showed that the patients in the IMV group frequently had cardiovascular or neurological diagnoses, greater disease severity on admission and a worse outcome. Yaman et al.^(20)^ showed that 93.8% of patients with NIV failure had underlying diseases.

Precise assessment of malnutrition and the provision of adequate nutritional support remain major challenges in patients admitted to ICUs, especially critically ill patients. In our study, we used two criteria for nutritional assessment-nutritional status and risk-using the Screening Tool Risk Nutritional Status and Growth (STRONGkids). As our sample consisted of 72.2% eutrophic children, no relationship or association was found between nutritional status and risk and the NIV outcome.

In a prospective study published in 2018 with adult patients, Dangers et al. found an association between dyspnea and NIV failure, specifically, the presence of dyspnea after the first session of NIV and not upon admission to the ICU, suggesting that dyspnea may be a marker to assess the response to NIV.^(28)^ Our retrospective study was limited by the lack of clinical variables such as dyspnea, vital signs, and laboratory test results.

In 2017, the PEMVECC reported that to avoid delayed tracheal intubation, NIV success must be evaluated up to 1 hour after its initiation by observing the following parameters: heart rate, respiratory rate, SpO_2_/FiO_2_, pH, the level of consciousness and the presence of organ failure.^(18)^

Limitations existed in our study, such as the retrospective design and the limited number of variables that may be related to the outcome, such as blood gas data and the initial ventilatory parameters. A strength of this study was our demonstration of the high effectiveness of NIV in children with respiratory failure. Over time, this practice has been strengthened in pediatric ICUs most likely due to greater clarification and training of a multidisciplinary team.

## CONCLUSION

A high success rate was observed with the use of noninvasive ventilation in the children with respiratory failure analyzed; those with a higher PRISM score at admission, comorbidities associated with the respiratory condition and using oxygen ≥ 40% have a higher risk of noninvasive ventilation failure.
